# Glucocorticoid Receptor Modulates EGFR Feedback upon Acquisition of Resistance to Monoclonal Antibodies

**DOI:** 10.3390/jcm8050600

**Published:** 2019-05-01

**Authors:** Valerio Gelfo, Francesca Pontis, Martina Mazzeschi, Michela Sgarzi, Maria Mazzarini, Rossella Solmi, Gabriele D’Uva, Mattia Lauriola

**Affiliations:** 1 Department of Experimental, Diagnostic and Specialty Medicine (DIMES), University of Bologna, 40138 Bologna, Italy; valerio.gelfo2@unibo.it (V.G.); martina.mazzeschi2@unibo.it (M.M.); michela.sgarzi@studio.unibo.it (M.S.); maria.mazzarini4@unibo.it (M.M.); rossella.solmi@unibo.it (R.S.); 2 Scientific and Technology Pole, IRCCS MultiMedica, 20138 Milan, Italy; francesca.pontis@multimedica.it (F.P.); gabriele.duva@multimedica.it (G.D.); 3 Centre for Applied Biomedical Research (CRBA), Bologna University Hospital Authority St. Orsola-Malpighi Polyclinic, 40138 Bologna, Italy

**Keywords:** epidermal growth factor receptor, glucocorticoid receptor, nuclear receptor, colon cancer, resistance to monoclonal antibodies, cetuximab (CTX), dexamethasone, mifepristone, positive feedback loops, negative feedback loops, IL-1A, IL-1B, IL-8, LRIG1, ERRFI1, DUSP1

## Abstract

Evidences of a crosstalk between Epidermal Growth Factor Receptor (EGFR) and Glucocorticoid Receptor (GR) has been reported, ranging from the modulation of receptor levels or GR mediated transcriptional repression of EGFR target genes, with modifications of epigenetic markers. The present study focuses on the involvement of EGFR positive and negative feedback genes in the establishment of cetuximab (CTX) resistance in metastatic Colorectal Cancer (CRC) patients. We evaluated the expression profile of the EGFR ligands TGFA and HBEGF, along with the pro-inflammatory cytokines IL-1B and IL-8, which were previously reported to be negatively associated with monoclonal antibody response, both in mice and patient specimens. Among EGFR negative feedback loops, we focused on ERRFI1, DUSP1, LRIG3, and LRIG1. We observed that EGFR positive feedback genes are increased in CTX-resistant cells, whereas negative feedback genes are reduced. Next, we tested the expression of these genes in CTX-resistant cells upon GR modulation. We unveiled that GR activation leads to an increase in ERRFI1, DUSP1, and LRIG1, which were shown to restrict EGFR activity, along with a decrease in the EGFR activators (TGFA and IL-8). Finally, in a cohort of xenopatients, stratified for response to cetuximab, we observed an inverse association between the expression level of LRIG1 and CRC progression upon CTX treatment. Our model implies that combining GR modulation to EGFR inhibition may yield an effective treatment strategy in halting cancer progression.

## 1. Introduction

Growth factors acting through receptor tyrosine kinases (RTKs) of ERBB family, along with steroid hormones acting through nuclear receptors (NRs), are critical signaling mediators of multiple cellular processes, including cell proliferation, survival, inflammation, differentiation, metabolism and migration [1]. For example, epidermal growth factor (EGF) binds EGF receptor (EGFR) and activates intracellular signaling cascades involved in the regulation of ductal and alveolar morphogenesis of the mammary gland [2]. Similarly, the activation of glucocorticoid receptor (GR) by the glucocorticoids, which belong to the steroid hormone family, controls cell proliferation during lobulo-alveolar development of the mammary gland [3] and promotes breast cancer metastasis [4].

Over the last 10 years, evidence of an emerging crosstalk between ERBBs and NRs have been reported, ranging from modulation of receptor levels and transcriptional activity to genetic and epigenetic markers [5]. ERBB-induced signalling has been proven to directly repress the expression of sex hormones (androgen and estrogen) receptors [6]. Otherwise, ERBB signaling can indirectly regulate glucocorticoid receptor expression e.g., through the Notch pathway in leukemic cells [7,8], or through the tyrosine kinase cKIT in human erythroblasts [9]. Further, activation of several ERBB downstream pathways, such as MAPK (ERK1/2) and phosphatidyl inositol 3-kinase PI3K/AKT axes, leads to an increase in estrogen receptor phosphorylation and transcriptional activity [10]. Notably, it has been reported that phosphorylation of oestrogen receptor α by PKA induces resistance to tamoxifen, a commonly used anti-oestrogen in breast cancer [11]. More recently, we delineated a robust regulation of EGFR signalling by GR activation, involving both a massive transcriptional repression of EGFR positive feedback loops and simultaneous production of the negative ones [5,12].

EGFR proved to be central to colorectal cancer (CRC) patients, who often present addiction to this pathway [13]. Thus, the EGFR pathway represents the major therapeutic target in CRC patients. Indeed, the administration of EGFR-directed monoclonal antibodies, such as CTX, has been shown to be a valuable treatment option in patients with advanced all-RAS-Wild Type CRC. Unfortunately, only a minority of patients achieves an objective response to this class of agents, and tumor regression is usually limited by the inevitable occurrence of drug resistance and therapy failure [14,15].

Genetic mechanisms, such as EGFR mutations or amplification and KRAS mutations, are leading causes of an unresponsive phenotype [16]. Furthermore, pathway bypass, signalling reactivation, and microenvironment-mediated cellular changes are emerging as an alternative mechanism in therapeutic resistance [16,17,18,19]. For example, microenvironment-secreted factors, such as cytokines and chemokines, protect tumor cells from death and support metastatic death spread [20]. In this regard, we previously reported that a panel of inflammatory cytokines, including IL-1A, IL-1B, and IL-8, induced by EGFR activation, is associated with impaired therapeutic efficacy in a cohort of xenopatients treated with CTX [21], and that patients unresponsive to CTX displayed higher levels of IL-1 receptor 1 (IL-1R1) [22].

The present study focuses on the involvement of EGFR positive and negative feedback genes in the establishment of CTX resistance in metastatic CRC patients. Among positive EGFR feedback regulators, we evaluated the expression profile of the EGFR ligands TGFA and HBEGF, which were previously found to be involved in CTX resistance [23,24], as well as the pro-inflammatory cytokines IL-1B and IL-8, which were negatively associated with monoclonal antibody response in xenopatients mice and patient specimens [21,25]. Among EGFR negative feedback loops we focused on ERRFI1, DUSP1, LRIG1, and LRIG3 [26]. We also tested the expression of these genes in CTX-resistant cells upon modulation of the Glucocorticoid Receptor (GR) activity.

We observed that EGFR positive feedback genes are increased in CTX-resistant cells, whereas negative feedback genes are reduced. We also unveiled that GR activation in CTX-resistant cells leads to an increase of EGFR negative feedback loops, which were shown to restrict EGFR activity, along with a decrease in the EGFR activators. In line with the data, we also demonstrated that GR activation is able to revert resistance to EGFR targeting agents. Our model supports the relevance of the GR-EGFR crosstalk in the context of CRC and the clinical exploit of this combination is discussed.

## 2. Material and Methods

### 2.1. Cell Culture

Caco-2 cells were maintained in DMEM supplemented with 10% fetal bovine serum and 1% Pen/Strep. For the establishment of CTX resistant Caco-2 clones (Caco-2 CXR) please refer to our previous work [21]. Caco-2 CXR were maintained in presence of cetuximab (Erbitux, Merck KgaA, Germany) at 10 µg/mL concentration. Caco-2 cells were authenticated by short tandem repeat (STR) profiling (PowerPlex 21 PCR Kit, Promega, Madison, WI, USA) and the certificate was released by the external service Eurofins Medigenomix Forensik GmbH (Ebersberg, Germany). Cells were routinely tested for mycoplasma contamination.

### 2.2. Colony Forming Assay

2.000 cells/well were seeded in 12-well plates, in 1 mL of medium. Treatments were added immediately or the following day, according to the information included in the figure legends. After ten days, the medium was removed, cells were washed with Phosphate-Buffered Saline (PBS) and fixed in paraformaldehyde (PFA) 4% for 20 min. After washing with PBS, cells were stained with crystal violet 0.5% for 30 min and then were washed with water to remove excess dye. A picture of each well was taken and the covered area was measured using ImageJ software. The mean value from covered area values returned by the software was calculated for each treatment and recorded as a percentage of control. Each experiment was repeated at least three times.

### 2.3. RNA Isolation and qPCR

RNA was extracted using TRIZOL^®^ Reagent (Invitrogen, Life Technologies, Monza, Italy) and resuspended in DEPC water. Total RNA quantity and quality were determined using a NanoDropTM Spectrophotometer (Thermo Fisher Scientific, Waltham, MA, USA). The RNA quality was then evaluated by 1% agarose gel electrophoresis and visualizing bands, stained with ethidium bromide, through a Geldoc transilluminator (Biorad, California, CA, USA). cDNA was synthesized using a retrotranscription mix consisting of buffer 5× (Thermo Fisher Scientific, Waltham, MA, USA), dNTP mix 1 mM, oligo dT 5 µM, random examer 5 µM, RiboLock RNase inhibitor 1 U/µL, RevertAid RT (Thermo Fischer Scientific, Waltham, MA, USA) 0,5 U/µL and water, and incubating for the reaction in a Thermal Cycler (VWR, Pennsylvania, PA, USA). Real-time qPCR analysis was performed with MaximaTM SYBR Green qPCR Master Mix 2X (Fermentas, Thermo Fisher Scientific, Waltham, MA, USA) in C1000TM Thermal Cycler (Bio-Rad, California, CA, USA).

The following primers were used (FH, forward; RH, reverse): FH_IL1B 5′-CTGAAAGCTCTCCACCTCCA-3′, RH_IL1B 5′-CCAAGGCCACAGGTATTTTG-3′. FH_IL8 5′-CGGAAGGAACCATCTCACTG-3′, RH_IL8 5′-AGCACTCCTTGGCAAAACTG-3′. FH_HBEGF 5′-GCTGTGGTGCTGTCATCTGT-3′, RH_HBEGF 5′-TCATGCCCAACTTCACTTTCT-3′. FH_TGFA 5′-GTTTTTGGTGCAGGAGGACA-3′, RH_TGFA 5′-CACCAACGTACCCAGAATGG-3′. FH_ERRFI1 5′-GGAATGAAAGCTACTGGTTG-3′, RH_ERRFI1 5′-GTTTTTAAACTCACTGCGAC-3′. FH_LRIG3 5′-TCGAATTGAACCGAAACAAG-3′, RH_LRIG3 5′-CCAAAAAGCTCCATCCATAAG-3′. FH_LRIG1 5′-AGAAGAGTGAAGAGTACAGTG-3′, RH_LRIG1 5′-CTGAGAAGAGAGGTAGCTTG-3′. qPCR signals (CT) were normalized to beta2-microglobulin (B2M), DDCT was calculated and each gene value was linearized to the time zero using the formula 2^-DDCT. Reaction efficiency (E) was calculated from the slope of the standard curve generated from 10-fold serial dilutions of calibrator cDNA, according to the formula E = (10 (−1/slope) −1) × 100.

### 2.4. Clustering and Heat-Map

The expression data of each gene, normalized on the B2M value, were organized in tab-delimited text files and loaded on the open source clustering software Cluster 3.0. Each column of data was normalized by center arrays function, which subtracts the column-wise median from the values in each column of data.

Median centered data were then analyzed and the unsupervised hierarchical clustering was generated by assuming a Euclidean distance as similarity metric, which takes into account the magnitudes of changes in the gene expression levels. Next, the clustering was followed by complete linkage and finally visualized as a heatmap using Java Treeview software (https://sourceforge.net/projects/jtreeview/) that allows interactive graphical analysis of the results from Cluster 3.0 [27].

### 2.5. Patients Data

LRIG1 gene expression information was based on a publicly available dataset of colorectal tumorgrafts from patients with wild type (WT) *KRAS*, *BRAF*, *NRAS*, and *PI3KCA* genotypes (“quadruple negative” tumors). WT quadruple negative tumorgrafts were tested for cetuximab response, as described by Bertotti and colleagues, who developed a molecularly annotated platform of patient-derived xenografts [28]. The data is currently available within the GEO databases (GSE76402). A comprehensive summary of the platform has been described by Isella and colleagues [29].

### 2.6. Statistical Analysis

The statistical analyses were performed using Prism version 6 (GraphPad Sotfware, www.graphpad.com/support/prism-6-updates/). Both t-test and one-way ANOVA were used to test the significance of the assays. The details of the applied statistical test are reported in the relevant figure legends.

## 3. Results

### 3.1. Generation and Validation of Caco-2 Cetuximab-Resistant Cells

**Figure 1:**
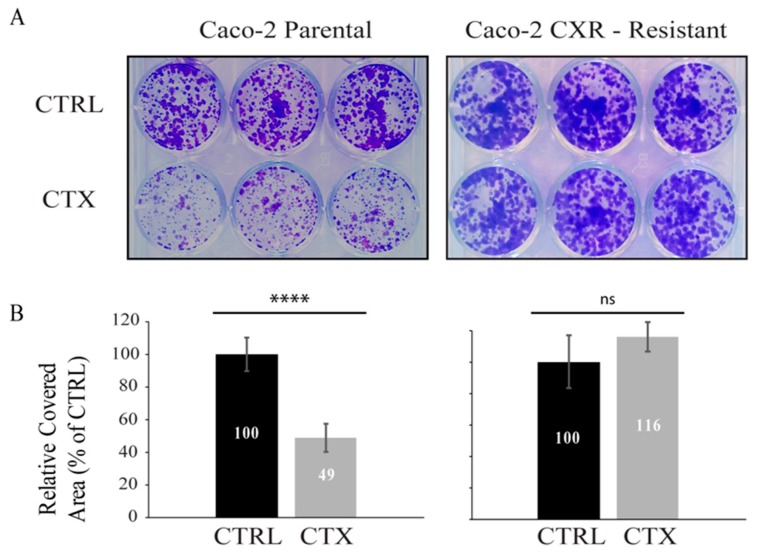
Colony forming assay of Caco-2 Parental and Caco-2 cetuximab resistant (CXR) clones. 2000 cells/well were plated with or without cetuximab (CTX) (10 µg/mL) for 10 days in DMEM medium supplemented with 10% fetal bovine serum, then cells were fixed, stained with Crystal Violet and quantified. Representative figures and quantification of the covered area by ImageJ are provided in A and B respectively. In (**A**) Caco-2 Parental cells (left) displayed 51% cell growth inhibition relative to CTRL, when treated with CTX whereas Caco-2 CXR–resistant cells (right) displayed undisturbed proliferation in the presence of CTX. (**B**) Quantification of relative covered area of Caco-2 Parental (left) and Caco-2 CXR–Resistant cells (right) in (A) by ImageJ is provided. The statistic was calculated by unpaired t-test, **** *p* < 0.0001, ns (not significant). This experiment was repeated more than three times.

In order to investigate the molecular mechanism implicated in the emergence of resistance to the monoclonal antibody CTX, we employed Caco-2 CTX-resistant (CXR) cells, a colorectal cancer in vitro model of resistance to CTX, recently established in our laboratory [21]. After ten days of CTX treatment, Caco-2 Parental cells appear partially responsive to CTX (Figure 1A, left), displaying a 51% cell growth inhibition relative to CTRL, as depicted in the quantification of Figure 1B. On the other hand, Caco-2 CXR displayed undisturbed proliferation under CTX treatment (Figure 1A), as reported in the quantification in Figure 1B, thus confirming the acquisition of CTX resistance.

### 3.2. EGFR Positive Feedback Loops are Increased in Cetuximab Resistant Cells

**Figure 2:**
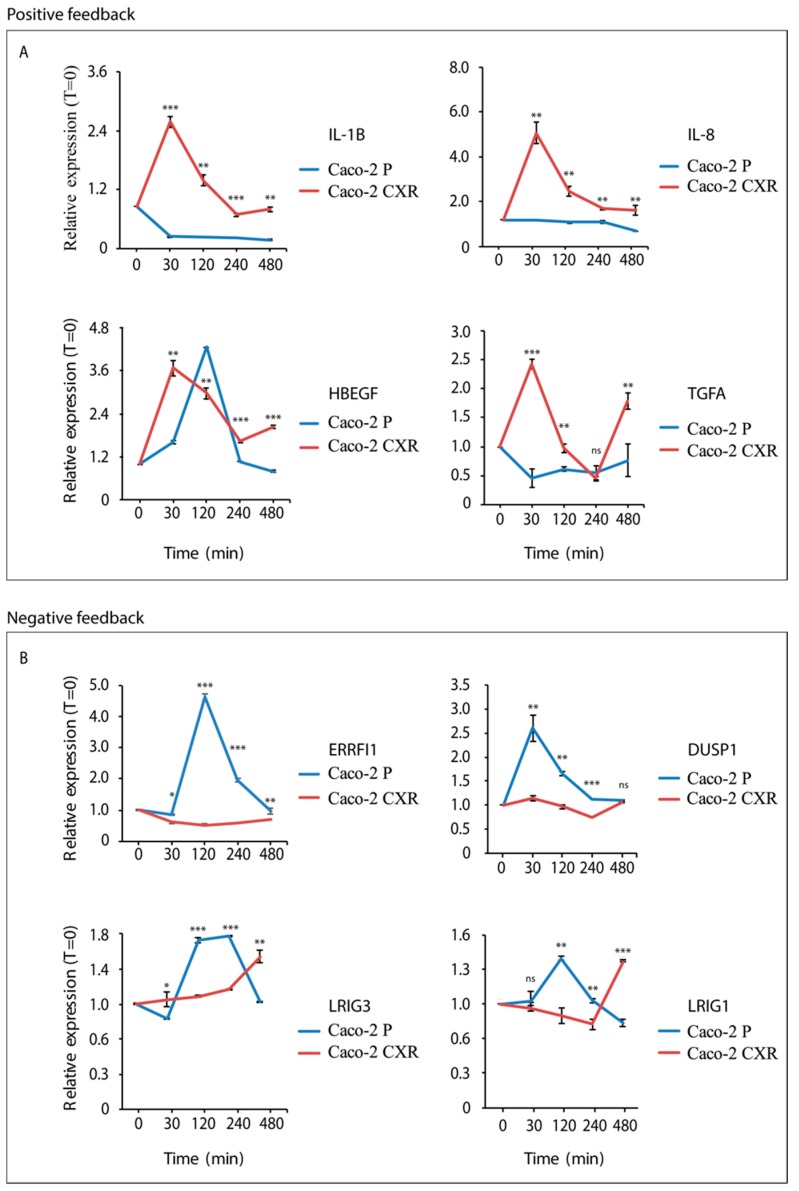
EGFR positive and negative feedback loops in Caco-2 Parental and Caco-2 cetuximab resistant (CXR) cell lines. Caco-2 Parental and Caco-2 CXR cell lines were treated in combination with cetuximab (CTX) (2 µg/mL) and EGF (20 ng/mL) at different time points (30, 120, 240 and 480 min) and qPCR analysis of EGFR positive (IL-1B, IL-8, HBEGF and TGFA) and negative (ERRFI1, DUSP1, LRIG1 and LRIG3) feedback genes was performed. In (**A**) EGFR positive feedback gene expression is reported. Caco-2 CXR activated immediate transcription of IL-1B, IL-8 and TGFA after 30 min from treatment and a decline of transcription level occurred after 4 hours, whereas Caco-2 Parental cells displayed non-significant changes in expression of IL-1B, IL-8 and TGFA. HBEGF displayed an overlapping profile in both Caco-2 Parental and CXR cell lines. In (**B**) EGFR negative feedback gene expression is reported. Caco-2 Parental cells showed an increase in the expression of ERRFI1, DUSP1, LRIG3 and LRIG1 genes, which was not observed in CXR cells. Statistical test of triplicated data was determined by multiple t-test, using the Holm-Sidak method, with alpha = 5%. Each row was analyzed individually, without assuming a consistent (standard deviation) SD. *** *p* < 0.0005, ** *p* < 0.01, * *p* < 0.05 and ns (not significant).

In order to shed light in the establishment of secondary resistance to CTX, we evaluated the expression profile probing EGFR positive and negative feedback loops. EGF-dependent transcriptional responses are characterized by early induction of auto-stimulatory loops comprising several growth factors such as TGFA and HBEGF, previously reported to be involved in CTX resistance [23,24] and interleukins such as IL-1B and IL-8, whose expression is predictive of the lack of response to EGFR targeting monoclonal antibodies, both in xenopatients treated mice and patient specimens [21,25]. On the other hand, negative EGFR feedback loops such as those involving LRIG1, LRIG3, ERRFI1 and DUSP1 proved to be inducible feedback inhibitors restricting EGFR activity [26]. For example, DUSP1 display a decreased expression in high histological grade tumors including prostate, colon, and bladder cancer [30]. In this work, we evaluated the gene expression of IL-1B, IL-8, TGFA and HBEGF in both Caco-2 Parental and Caco-2 CXR cells undergoing CTX and EGF stimulation at different time points (30, 120, 240 and 480 min). Our data show that the combination of CTX and EGF stimuli in CTX resistant cells activates immediate transcription of IL-1B, IL-8, and TGFA genes, already after 30 min, whereas no induction was recorded in Parental cells (Figure 2A). mRNA levels of these genes display a decay after 2 hours, probably due to a process of active mRNA degradation, as previously described [31]. On the other hand, HBEGF displayed an overlapping expression profile in both Caco-2 Parental and CXR cells treated as above described (Figure 2A). Next, we evaluated the production of ERRFI1, DUSP1, LRIG1 and LRIG3. Following CTX and EGF combined stimuli, Caco-2 Parental cells displayed a sharp increase in the expression of ERRFI1, DUSP1, LRIG1, and LRIG3, which was not observed in CTX resistant cells (Figure 2B). Altogether, these observations support an opposite profile in mRNA production in EGFR positive and negative feedback, when tested in CTX sensitive or resistant cells.

### 3.3. Glucocorticoids Administration Downregulates EGFR Positive Feedback Loops and Boosts EGFR Negative Feedback Loops in Cetuximab-Resistant Cells

Our data thus far demonstrated that CTX plus EGF treatment strongly promotes EGFR positive feedback and decreases EGFR negative feedback loops in Caco-2 CTX-Resistant cells. In that regard, we previously demonstrated in normal breast cells that activation of GR suppresses EGFR’s positive feedback loops and simultaneously triggers negative feedback loops, thus terminating EGFR signalling [12]. Indeed, in the MCF10A cell line, treatment with glucocorticoids activate expression of ERRFI1/MIG6 [12], which physically bind EGFR kinase domain leading to receptor inactivation [32]. Further, glucocorticoids are well known anti-inflammatory agents that suppress the expression of pro-inflammatory cytokines (including IL-1B and IL-8) through NF-kB inhibition [33].

**Figure 3:**
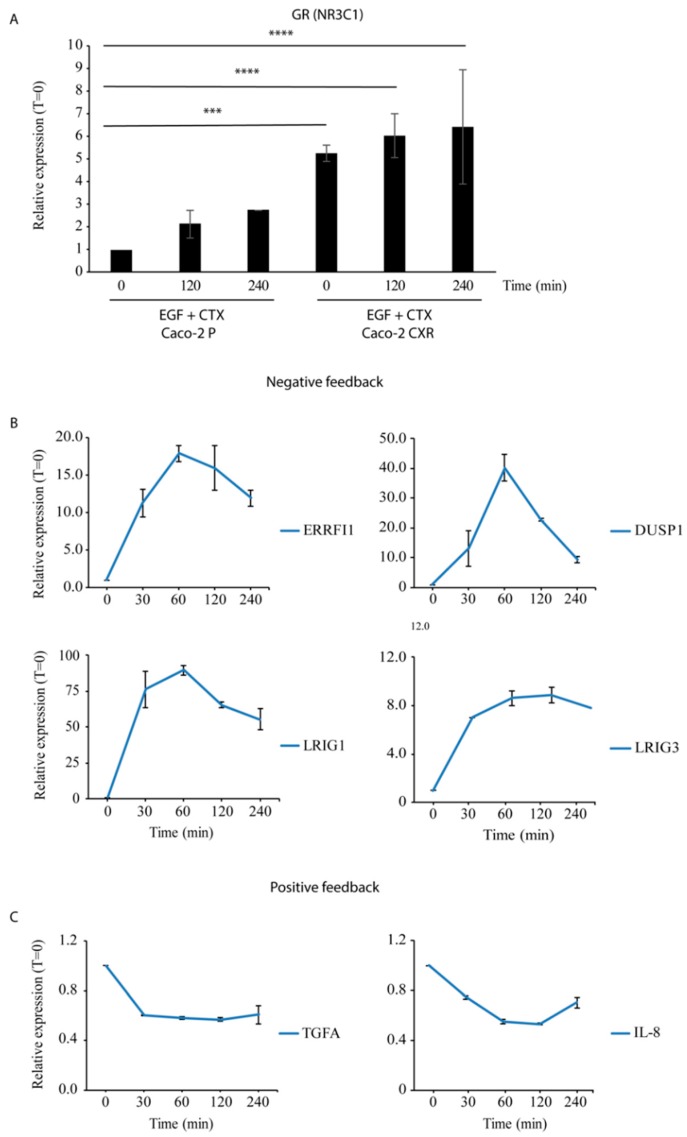
Dexamethasone suppresses EGFR positive regulators and boosts negative feedback loops in cetuximab (CTX) resistant cells. (**A**) Caco-2 Parental (P) and Caco-2 cetuximab resistant (CXR) cells were treated with cetuximab (CTX, 2 µg/mL) in combination with EGF (20 ng/mL) for 120 and 240 min. Notably, Caco-2 CXR displayed a higher basal level of GR mRNA compared to Caco-2 P counterpart. We also observed an increase in GR mRNA production in both Caco-2 P and Caco-2 CXR, compared to time 0. The ordinary one-way ANOVA with Dunnett’s multiple comparisons test was applied to evaluate statistical changes in relation to Parental cells to time 0. **** *p* < 0.0001 and *** *p* < 0.0005. (**B**) Caco-2 CXR cells were pre-treated with dexamethasone (DEX, 1 µM) for 10 min, and stimulated with a combination of CTX (2 µg/mL) and EGF (20 ng/mL) at different time points (30, 60, 120 and 240 min). qPCR analysis was performed in triplicate for EGFR negative feedback loops (ERRFI1, DUSP1, LRIG3 and LRIG1). mRNA levels displayed a strong increase after 30 min of stimulation, with the maximum hit after 60 min. Apart from DUSP1, high expression level of these genes was maintained over the time course (up to 8 hours). (**C**) mRNA levels of EGFR positive feedback genes (TGFA and IL-8) are depicted. Following DEX stimulation, a decrease in the mRNA production of TGFA and IL-8 was observed already at 30 min and was maintained all over the time course.

We first evaluated GR (NR3C1) expression in Caco-2 Parental and Caco-2 CXR cells after 120 and 240 min of CTX treatment in combination with EGF (Figure 3A). Time 0 of Caco-2 Parental cells was considered as CTRL, with no EGF and CTX treatment. Notably, Caco-2 CXR displayed a higher basal level of GR mRNA compared to Caco-2 Parental counterpart (Figure 3A). Following CTX and EGF treatments, we also observed an immediate (after 120 and 240 min) slight increase in mRNA production of GR in both Caco-2 Parental and Caco-2 CXR compared to CTRL.

Next, we evaluated the effect of the synthetic GR agonist Dexamethasone (DEX), in combination with CTX and EGF, on mRNA production of TGFA, IL-8, ERRFI1, DUSP1, LRIG3, and LRIG1 over a four-hour time course in Caco-2 CXR cells. Upon stimulation with DEX, our results show an immediate burst of ERRFI1, DUSP1, LRIG1 and LRIG3, which was detected after 30 min and reached the maximum hit after 60 min (Figure 3B). Notably, DUSP1 displayed a forty folds induction upon DEX treatment with a rapid decay after 240 min, probably due to increased mRNA degradation. On the other hand, ERRFI1, LRIG1 and LRIG3 expression levels remained sustained even at 240 min of stimulation. The opposite trend was observed for the mRNA production of positive feedback genes (TGFA and IL-8), which appeared inhibited by DEX treatment, already after 30 min of stimulus (Figure 3C).

### 3.4. GR Modulation Re-Activates EGFR Negative Feedback Loops in Cetuximab-Resistant Cells

**Figure 4:**
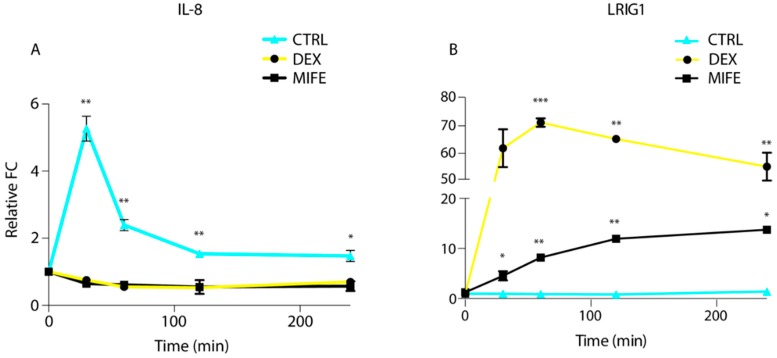
Comparison of gene expression level of EGFR positive (IL-8) and negative (LRIG1) feedback loops following dexamethasone (DEX) and mifepristone (MIFE) treatment in cetuximab resistant (CXR) resistant cells. Caco-2 CXR cells were pretreated with DEX (1 µM) or MIFE (1 µM) and stimulated with a combination of cetuximab (CTX,2 µg/mL) and EGF (20 ng/mL) at different time points (30, 60, 120 and 240 min). qPCR analysis was performed for IL-8 and LRIG1 feedback loops. (**A**) IL-8 gene expression level following DEX or MIFE treatment shows an immediate decrease in gene expression (after 30 min), maintained during the whole-time (up to 8 hours) compared to time 0. CTRL showed a five folds increase in gene expression level after 30 min from CTX and EGF treatment. (**B**) Increase in LRIG1 gene expression following DEX or MIFE treatment. Notably, Caco-2 CXR following DEX treatment displayed a burst in LRIG1 expression with seventy folds gene expression increase when compared to time zero. Statistical test of triplicated data was determined by multiple t-test, using the Holm-Sidak method, with alpha = 5%. Each row was analyzed individually, without assuming a consistent SD. *** *p* < 0.0005, ** *p* < 0.01, * *p* < 0.05.

To further investigate the interactions between EGFR and glucocorticoid receptor signalling in the context of resistance to CTX, we employed the GR antagonist mifepristone (MIFE), which is able to dampen the receptor activity, but acts as partial agonist in absence of GR ligands [34]. We selected IL-8 and LRIG1 to test the gene expression levels, in the presence of MIFE or DEX. IL-8 expression results in a five-fold increase after 30 min of stimulation with the CTX and EGF combination (Figure 4A). Interestingly, administration of DEX and MIFE resulted in a decrease of IL-8 gene expression in resistant cells (Figure 4A), suggesting a partial agonistic activity of MIFE in these cells and settings.

Consistently with our previous results, LRIG1 expression under CTX plus EGF stimulation did not display any increase in resistant cells (Figure 4B), while GR activation by DEX strongly enhanced the production of LRIG1, otherwise barely expressed. Notably, we recorded a seventy-fold increase in gene expression level for LRIG1 after one hour of DEX treatment when compared to time zero (Figure 4B), while MIFE displayed a milder, but still consistent ten folds LRIG1 production. Taken together, these results show an impairment in GR modulation in the context of cetuximab resistance, and that EGFR negative and positive feedback loops are under the control of the glucocorticoid receptor.

**Figure 5:**
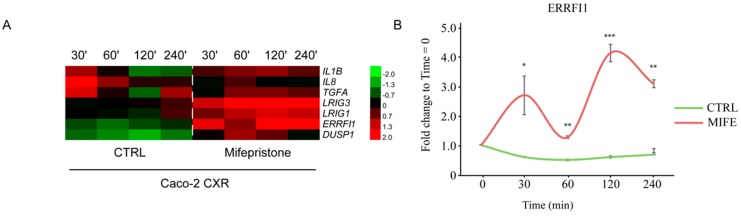
Inhibition of glucocorticoid receptor reactivates negative feedback loops in cetuximab-resistant Caco-2 cell line. (**A**) Heatmap showing gene expression of EGFR positive (IL-1B, IL-8 and TGFA) and negative (LRIG3, LRIG1, ERRFI1 and DUSP1) feedback loops, in Caco-2 cetuximab resistant (CXR) cells pretreated with and without mifepristone (MIFE, 5 µM) for 10 min and stimulated with a combination of cetuximab (CTX, 2 µg/mL) and EGF (20 ng/mL) at the indicated time points (30, 60, 120 and 240 min). (**B**) ERRFI1 gene expression profile over time following treatments described in A. Statistical test of triplicated data was determined by multiple t-test, using the Holm-Sidak method, with alpha = 5%. Each row was analyzed individually, without assuming a consistent SD. *** *p* < 0.0005, ** *p* < 0.01, * *p* < 0.05.

Next, we performed a comprehensive analysis of IL-1B, IL-8, TGFA, LRIG1, LRIG3, ERRFI1 and DUSP1 over a time course. Resistant cells were pretreated with MIFE and then stimulated with a combination of CTX and EGF at different time points (30, 60, 120, and 240 min). Upon CTX and EGF stimulation, we confirmed a swift burst of TGFA and interleukins IL-1B and IL-8 followed by a fast decay, while under MIFE pretreatment these genes showed a delayed milder production (Figure 5A). On the other hand, MIFE pretreatment boosted the transcription of LRIG1, LRIG3, ERRFI1, and DUSP1, which appeared sustained up to four hours (Figure 5A). In Caco-2 CXR, pretreated with MIFE and stimulated with a combination of CTX and EGF, ERRFI1 displayed a gene expression activation characterized by a specific pattern, with two peaks of expression at 30 and 120 min, as displayed in Figure 5B. Thus, GR antagonist MIFE induces a bimodal activation of ERRFI1, which was not observed in MIFE untreated cells after 4 hours of CTX and EGF administration (Figure 5B). To summarize, modulation of GRs reinstates EGFR negative feedback loops in the context of CTX resistance.

### 3.5. LRIG1 Expression Predicts Resistance to Cetuximab in Colorectal Cancer Patients

**Figure 6:**
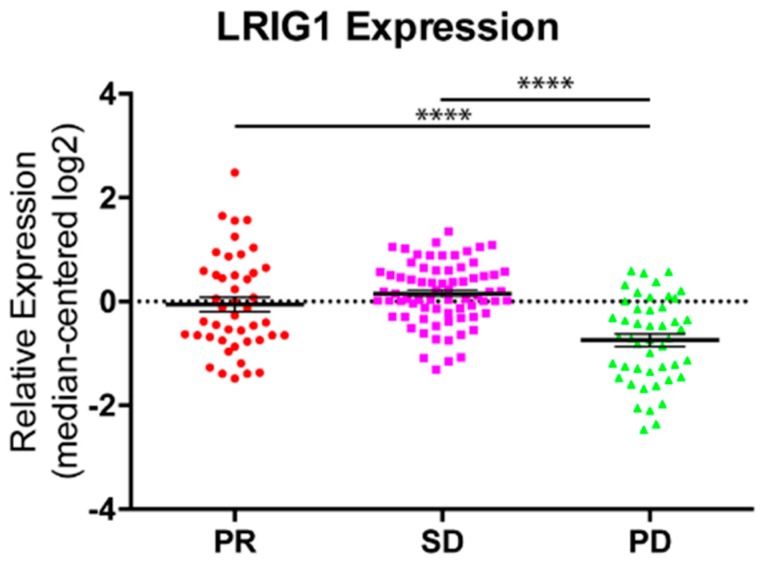
*LRIG1* expression predicts resistance to cetuximab in colorectal cancer xenopatients. LRIG1 expression levels were evaluated in a cohort of CRC patients derived xenografts quadruple wild type for *KRAS*, *BRAF*, *NRAS* and *PIK3CA*, subdivided by response to cetuximab therapy: disease regression (PR), disease stabilization (SD), disease progression (PD). Ordinary one-way ANOVA was performed, with Tukey’s multiple comparisons test. **** *p* < 0.0001.

Our data in vitro pointed out a defect in the production of both inflammatory cytokines and EGFR negative feedback genes, in the context of cetuximab resistance. We therefore decided to analyze a dataset from 98 patients with wild-type *KRAS*, *BRAF*, *NRAS*, and *PIK3CA* genotypes (“quadruple negative” tumors). WT quadruple negative tumorgrafts were tested for cetuximab response, as previously described [28,29]. In this condition, the human stroma is substituted by murine components; therefore, this analysis covers only receptors and autocrine ligands expressed by cancer cells. Interestingly, we observed an inverse association between the expression level of the EGFR negative regulator LRIG1 and CRC progression upon treatment (Figure 6). Indeed, in accordance with the in vitro data, LRIG1 displayed a decreased expression in patient derived xenograft that proved to be resistant to EGFR blockade (tumor volume increase of at least 35% compared to the initial pre-treatment volume). No differences were detected in the two groups with limited sensitivity to cetuximab, with tumor volume changes between 35% increase and 50% reduction, which is considered a stable disease (SD). Furthermore, no association was observed for ERRFI1, DUSP1 and LRIG3 (data not shown), whereas a positive association for IL-8 and IL-1B was described in our previous manuscript [21,22].

These results suggest that gradual tumor adaptation to EGFR blockade might be associated with downregulation of EGFR negative regulator, LRIG1, which might be responsible for an unstrained EGFR inhibition.

## 4. Discussion

During the last fifty years, the ErbB receptors family has emerged as key regulators of critical cellular processes such as proliferation, differentiation, cells survival, migration and cell cycle control [35]. The integration and often intertwining of negative and positive feedback loops help maintaining appropriate quantitative and dynamic relationships between inputs (growth factor stimuli) and outputs (cellular phenotype). Therefore, feedback loops provide precision, robustness, and versatility to intercellular signals [36,37]. Collectively, the regulatory loops dictate the duration, amplitude, and frequency of signals [36], allowing fast and stable attainment of the steady state [38].

Deregulation of positive and negative feedback loops proved to be implicated in diverse types of human cancers, primarily in carcinomas of secretory epithelia [39]. For example, autocrine production of the EGFR ligand TGFA was confirmed to be linked to an increased risk of developing metastasis in colorectal cancer [40], whereas a feedback activation of EGFR was responsible for the failure of BRAF inhibition in patients carrying the mutation BRAF(V600E) [41].

We recently showed an antagonistic interaction between the EGFR pathway and the glucocorticoids receptor, involving a group of feedback modifiers of signal transduction, demonstrating that GCs inhibit positive feedback loops (TGFA and HBEGF) activated by growth factors, while simultaneously stimulating the reciprocal inhibitory loops (DUSP1 and ERRFI1) [1,12].

Our data show a remarkable difference between CTX unresponsive and responsive cells regarding EGFR positive and negative feedback loops regulation. CTX treatment in sensitive cells induces an increase of negative feedback loops (ERRFI1 and DUSP1) and the partial inhibition of positive feedback loops (IL-8, IL-1B and TGFA). In contrast, in CTX resistant conditions, the expression profile displays an opposite trend upon CTX treatment, characterized by an induction of IL-1B, IL-8 and TGFA, along with inhibition of negative feedback loops. In this regard, TGFA was previously found to be involved in the acquisition of CTX resistance in vitro as its overexpression induced the EGFR-MET interaction leading to the activation of downstream MET effectors PI3K/AKT and ERK activation [23,24].

Production of negative feedback loops proved to reduce the amplitude and markedly shortens the duration of EGF-induced ERK activation intercepting the RTK-to-ERK signalling at multiple levels. On the other hand, positive feedback loops prolong and amplify the response to a weak signal and increase the sensitivity of the system to signalling inputs by amplifying the stimulus [42,43]. Upon resistance to EGFR inhibition by monoclonal antibodies, cancer cells display a rewiring of feedback production, with an upregulation of genes that are able to sustain the signal and a downregulation of genes intrinsically able to dampen the EGFR pathway.

**Figure 7:**
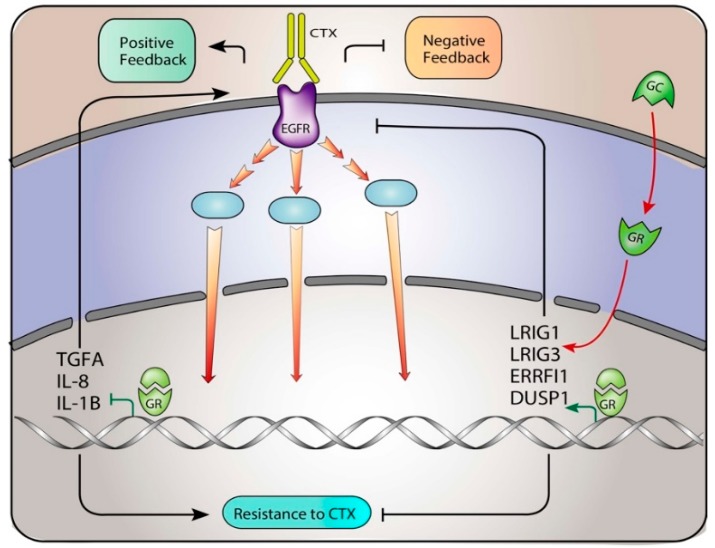
A model depicting the rewiring of resistance to cetuximab in colorectal cancer xenopatients. EGFR positive feedback loops are increased in cetuximab resistant cells that under cetuximab (CTX) stimulus displayed increased abundance of IL-8, IL-1B, TGFA and HBEGF, along with the dampened production of LRIG1, LRIG3, ERRFI1 and DUSP1. GR modulation re-activates EGFR negative feedback loops in cetuximab-resistant cells.

Here, we show that the receptor for glucocorticoids modulates EGFR pathway, by mean of inhibition of the growth factors TGFA and HBEGF and by simultaneous stimulation of the inhibitory genes: DUSP1, ERRFI1, LRIG1 and LRIG3 [1,12]. A graphical summary is depicted in Figure 7.

In line with this concept, our data showed that upon acquisition of resistance to CTX, EGFR pathway feedback loops display an alteration compared to control cells, which could be reversed by administration of the steroid hormone dexamethasone. These data are relevant also for patients, where LRIG1 was found to be negatively associated to CTX response, while IL-8 and IL-1B were positively associated [21,22].

Although both ERBB and GRs pathways have been extensively explored, little is known about their possible systematic interaction in the context of drug resistance. Deeper understanding of EGFR-GR crosstalk is critical, mainly because this combination is already employed in ongoing clinical treatments. Indeed, often steroid hormones represent a widely used co-medications in clinical settings. The evidence that GR modulates EGFR pathway in the context of cetuximab resistance offers the basis for new routes of pharmacological intervention in otherwise untreatable patients.

## 5. Conclusions

To summarize, we conclude that an upregulation of positive feedback loops and a repression of negative feedback loops in CTX resistant tumors could lock EGFR pathway in an activate state, thus escaping feedback control and guiding cells toward a surviving and proliferating phenotype. In addition, our data demonstrated that modulation of GRs receptor in vitro, in a context of CTX resistance, may dampen positive feedback loops and reinstate negative feedback loops. Thus, GR modulation may represent a successful strategy to improve cetuximab efficacy in CRC patients.
